# Plants with Therapeutic Potential for Ischemic Acute Kidney Injury: A Systematic Review

**DOI:** 10.1155/2022/6807700

**Published:** 2022-05-23

**Authors:** Arif Ali, Tiago Lima Sampaio, Haroon Khan, Philippe Jeandet, Esra Küpeli Akkol, Humaira Bahadar, Alice Maria Costa Martins

**Affiliations:** ^1^Postgraduation Program in Pharmacology, Federal University of Ceará, Fortaleza, Brazil; ^2^Department of Clinical and Toxicological Analysis, Federal University of Ceará, Fortaleza, Brazil; ^3^Department of Pharmacy, Abdul Wali Khan University, Mardan 23200, Pakistan; ^4^University of Reims Champagne-Ardenne, Research Unit Induced Resistance and Plant Bioprotection, USC INRAe 1488, SFR Condorcet FR CNRS 3417, Faculty of Sciences, Reims 51687, France; ^5^Department of Pharmacognosy, Faculty of Pharmacy, Gazi University, Etiler, Ankara 06330, Turkey; ^6^Department of Biochemistry and Molecular Biology, Federal University of Ceará, Fortaleza, Brazil

## Abstract

Acute kidney injury (AKI) is a complex condition which has an intricate pathology mostly involving hemodynamic, inflammatory, and direct toxic effects at the cellular level with high morbidity and mortality ratios. Renal ischemic reperfusion injury (RIRI) is the main factor responsible for AKI, most often observed in different types of shock, kidney transplantation, sepsis, and postoperative procedures. The RIRI-induced AKI is accompanied by increased reactive oxygen species generation together with the activation of various inflammatory pathways. In this context, plant-derived medicines have shown encouraging nephroprotective properties. Evidence provided in this systemic review leads to the conclusion that plant-derived extracts and compounds exhibit nephroprotective action against renal ischemic reperfusion induced-AKI by increasing endogenous antioxidants and decreasing anti-inflammatory cytokines. However, there is no defined biomarker or target which can be used for treating AKI completely. These plant-derived extracts and compounds are only tested in selected transgenic animal models. To develop the results obtained into a therapeutic entity, one should apply them in proper vertebrate multitransgenic animal models prior to further validation in humans.

## 1. Introduction

Acute kidney injury (AKI) is a widely spread and prospectively life-taking disease. Presently, AKI definition is based on the decline in the kidney function in more or less than a week [1]. Based on AKI genesis, this disease has been characterized into three categories, prerenal—a physiologic reaction of the normal structural kidney towards hypoperfusion; intrinsic or intrarenal—injury to the kidney parenchymal cells; and postrenal—a response towards a urinary tract obstruction [2]. The impact of AKI in long run on public health is enormous. AKI is associated with increased number of morbidity and mortality. The incidence of AKI has almost doubled in number over the past two decades [3, 4]. The inpatients are more likely to suffer from AKI as a result of secondary disease complications or due to an adverse reaction to therapy. The prevalence of AKI among seriously ill patients is 25–67%, while the mortality ratio is 30–60% even if the severity of the disease subsides [5–8].

Annually, about 2100 per million of the population suffer from AKI. In developed countries, the cases of AKI are expected to be more than 2 million per annum. Among these, 1.5 million patients survive of which many patients progress to advanced stages of chronic kidney disease (CKD) within a period of 24 months. To these numbers, one can add over 300,000 patients to the category of advanced stage of CKD per year. The episodes of AKI in CKD patients lead to upsurge in the development of end-stage renal disease (ESRD). These figures provide the fact that the major attributable risk in patients suffering from CKD is AKI [1].

AKI is a multifactorial condition, and the pathogenesis of which is based on hemodynamic, inflammatory, and direct toxic effects at the cellular level [9]. However, renal ischemic reperfusion injury (RIRI) is being considered as one of the foremost reasons of AKI which is accompanied by 50% mortality ratio in intensive care units [10–12]. Renal ischemic reperfusion injury may occur due to various reasons such as administration of vasoconstrictive drugs or radio-contrast agents. Hypotension also leads to RIRI which more commonly occurs in sepsis or when a large volume of fluid is lost in trauma. Similarly, it can be initiated by various clinical ailments like myocardial infarction, different types of strokes, or due to postsurgical operations such as organ transplant, cardiac surgery, extracorporeal lithotripsy, and adrenal aneurysm. In case of trauma or shock, the body compensates fluid losses by various mechanisms, but due to unmet need of high oxygen demand of cells and insufficient metabolic substrate availability, the cellular injury often leads to organ failure [13–15].

Literature data prove the hypothesis that acute kidney injury due to ischemic reperfusion is associated with changes in hemodynamics and dysfunction of endothelial cells because of a high level of reactive oxygen species (ROS) and reactive nitrogen species (RNS) leading to decreased production of nitrogen oxide and intracellular energy store exhaustion. Both ROS and RNS stresses cause lipid peroxidation, oxidative DNA damage, modification of inflammatory pathways, modification of leukocyte function, and microvascular reduction in blood flow to renal medulla because of vascular congestion [10]. Similarly, Malek and Nematbakhsh have mentioned that ROS is involved in kidney injury by lipid peroxidation, while oxidative damage of proteins and DNA contributes to apoptosis and cell necrosis. Downregulation of antioxidant enzymes such as catalase, superoxide dismutase (SOD), and glutathione peroxidase (GPx) might be responsible for the pathophysiology of the ischemic reperfusion injury [16].

The most efficient and effective ways to prevent ischemic reperfusion (I/R) injury is thus to scavenge these ROS and free radicals. According to current scientific data, traditional natural medicines have been found very effective against ROS and oxidative stress. Moreover, natural therapies and mixtures have been shown to be very efficacious in various inflammatory conditions [17]. The purpose of this review is to summarize the treatments used for AKI induced by I/R injury via plant extracts and plant-derived natural compounds, thereby offering some clue for further in-depth research.

## 2. Materials and Methods

Relevant articles were retrieved from various database search engines such as Google Scholar, PubMed, and ScienceDirect by typing key terms “acute kidney injury,” “renal ischemic reperfusion in rats,” “renal ischemia reperfusion,” “renal ischemic reperfusion and plant extracts,” “nephroprotection against renal/kidney with renal. reperfusion,” and “plant extracts against kidney ischemia reperfusion.” Articles published from 2000 to 2019 were selected to write this review.

After careful reading of the articles, the central information was critically collected and discussed according to the following thematic sessions: kidney with renal. reperfusion injury, epidemiology, etiology of AKI, diagnosis, treatment of AKI, mechanisms by which plants ameliorated renal I/R injury induced-AKI, and effects on biomarkers and renal tissues. The final curatorship of the articles took place through the tabulation of relevant information such as substances, sources, experimental models, methodological aspects, mechanisms, and effects.

## 3. Results

### 3.1. Renal Ischemic Reperfusion Injury

Ischemic reperfusion (I/R) injury is a pathophysiological condition which is generated by preliminary impediment of blood flow to an organ followed by restoration or reperfusion. The duration and extent of ischemia govern the range of cell death or loss of organ function. The reperfusion phase is thought to reinstate oxygen and nutrient requirement of an organ though it also synergizes the oxidation and inflammatory stress both locally and systemically resulting in cell damage. This whole process is being entitled as I/R injury [18].

Renal I/R injury causes hypoxia as a result of which metabolism is altered leading to depletion of adenosine triphosphate (ATP) and increase in lactate concentration. This provokes an electrolyte imbalance such as an increased sodium level as well as a water influx and an intracellular calcium overload. As a result, an increase in apoptosis besides cell necrosis and intracellular acidosis is observed. Furthermore, reperfusion leads to generation of ROS decreasing SOD and catalase as well as GPx levels. Reperfusion inhibits the cytochrome c oxidase and increases NO levels. These parameters are responsible for protein and lipid peroxidation (which increases MDA levels) along with DNA damage, which further aggravates cell necrosis and apoptosis [19, 20]. Because of the redox imbalance, a local and systemic inflammatory process is activated. I/R injury is also responsible for the initiation of several inflammatory reactions within the renal parenchyma. In addition to infiltration of neutrophils, renal I/R injury is accountable for the generation of many inflammatory cytokines, for instance IL-6, IL-1, and TNF-*α* [21].

In experimental renal I/R injury models, the renal blood flow is completely blocked by clamping the renal artery. This blocking phase is called ischemia, the duration of which is usually from 20 to 60 minutes, and this phase is accompanied by hypoxia in addition to loss of the GFR function. Within the first 30 minutes of ischemia, the injury is still limited, but within 45 minutes, the injury becomes more regular and confluent with different levels of necrotic lesions. After 60 minutes, necrotic cells become infarcted [22–25]. A total blockage during ischemia results into endothelial cell injury with functional loss. It also results in substantial modifications of the transcription program of vasoactive cytokines and leukocyte function [26]. Different approaches are in practice for the induction of renal ischemia in experimental models, such as the simultaneous application of bilateral renal ischemia [27, 28], while others apply unilateral ischemia in one kidney and perform nephrectomy of the other [29, 30].

Removal of clamps and restoration of blood supply and nutrients is called reperfusion. This rapid resupply of blood is essential for the survival of cells, which are being damaged by ischemia. However, reperfusion, which is accompanied by a return of oxygen, itself is the major step responsible for further cell injury [20]. The foremost damaging effect of reperfusion is the generation of ROS via mitochondria, which are responsible for acute injury to cells, persisting for weeks without any intervention. ROS causes a disturbance in adenosine triphosphate (ATP) production and calcium regulation leading to mitochondrial permeability transition pore (MPTP) opening. This dysregulation initiates cell necrosis and apoptosis as well as cell death [31].

Various sources have been linked with ROS generation following I/R injury such as enzymatic sources including xanthine oxidase, NADPH oxidase, the mitochondrial electron transport chain, and nitric oxide synthase [32]. The ROS generated from these sources by I/R injury highly target proteins, cell membrane lipids, and nucleic acids [33]. Besides the direct cytotoxic effect of renal I/R injury, ROS also prompts an inflammatory response in renal endothelial and parenchymal cells. As a result, many proinflammatory cytokines such as the tissue necrosis factor-*α* (TNF-*α*), interleukins (IL) 1, IL-6, and many chemokines like the monocyte chemoattractant protein (MCP) and IL8 are released. The ROS and cytokines generated as a result of kidney injury induce an upsurge in the expression of adhesion biomarkers, for instance, the intercellular adhesion molecule (ICAM), the vascular cell adhesion molecule (VCAM), and the P-selectin [21, 34]. Assembling of cytokines, chemokines, ROS, and adhesion molecules further aggravate the renal injury.

### 3.2. Clinical Presentation of AKI

#### 3.2.1. Epidemiology

Accurate elucidation of AKI epidemiology is modulated by many factors such as various definition of AKI and variations in case mingling. For instance, patients of non-ICU cases are different from ICU patients. Similarly, there is a difference between cases in general hospital ICU compared to rural hospital ICU; postoperative cardiac patients are different from those displaying liver cirrhosis [35]. Many factors are involved in the variation of AKI figures, in developed as well as in developing countries [36]. Aged populations are more affected by AKI in developed countries [37], while in developing countries, mostly adults and children suffer from AKI due to socioeconomic and environmental influences [38]. Studies involving adult population have revealed that AKI is accompanied by an increased rate of mortality, hospital ICU stay, and dependency on mechanical ventilation [39–41]. AKI commonly occurs in hospitalized patients and more frequently in those who are admitted in ICU [39, 42]. The prevalence of AKI in ICU admitted patients is mentioned in various epidemiological studies in accordance with KDIGO criteria [39, 43, 44]. A study performed by Kaddourah et al. included children and young adults having an age range from 3 months to 25 years. The total number of patients registered were 4683, admitted in 32 ICUs. During the first week of admission at the hospital, the reported AKI incidence was 27%, of whom 12% developed severe AKI [45].

The ratio of AKI in hospitalized pediatric patients is about 5%, while in seriously ill pediatric patients, it ranges between 20 and 70% accompanied by high morbidity and mortality rates [46, 47]. A systematic review based on 312 cohort studies from high-income developed countries comprising 49 million patients of AKI has shown that one in five adults suffered from AKI. Likewise, one in three children were displaying AKI. Another cohort study (*n* = 5, 23,390) performed in Scotland and based on the RIFLE classification system reported that the incidence of AKI was 1,811 per million residents [48]. The prevalence of AKI was also studied in 120,123 patients from January 2000 to December 2005 in 57 ICUs across Australia [49], the number of AKI cases mentioned being 36%. According to the RIFLE classification of AKI, most of the cases (16%) belonged to the “R” category (stage I injury), 13.6% were of “I” (stage II injury), and 6.3% cases were related to the “F” category (stage III injury). The AKI was associated with an increased mortality ratio [49]. Concerning AKI prevalence in ICUs, a study was conducted in Thailand including 5,377 patients from February 2013 till July 2015. Among them, 2471 (53%) patients were diagnosed with AKI during hospital admission [50]. Another study from Scotland has shown a prevalence of 2147 AKI cases per one million residents [48]. Most of the aforementioned studies have thus reported a sovereign link of AKI with a greater risk of mortality. The worldwide projected mortality rate of AKI is 24% in the adult population and 13.8% in children. Among aged population, the risk of mortality from AKI is even higher [51].

#### 3.2.2. Etiology of AKI

There is a difference in the susceptibility of each individual to develop AKI caused by exposure to numerous factors depending, for instance, on the duration of the exposure, its type, heterogeneity, and severity [52]. As stated before, AKI is more common in critically ill patients and its cause is frequently multifactorial. Sepsis is one of the serious conditions that is increasing in hospitalized patients. For example, a 22-year analysis of hospitalized patients carried out in the U.S has revealed a yearly growth of 8.7% in sepsis diagnosis [53]. Sepsis has been found as the foremost cause of AKI accounting for 45 to 70% of the cases of AKI being linked to sepsis. A large prospective observational study performed by Bagshaw et al. has mentioned that, among 29,000 patients, 5.7% developed AKI of which 47.5% cases were due to sepsis [54]. Likewise, pediatric studies showed that sepsis was the major risk factor in 18 to 58% of the patients acquiring AKI [55].

Major surgery is another factor leading to AKI, as revealed by Grams et al. (2016) according to a large cohort study involving 161,185 participants. Among the participants, 11.8% suffered from AKI after a major surgery, though other risk factors in these patients were old age, male gender, overweight, and African American race. There were differences in the prevalence of AKI with respect to the type of surgery, but the most affected ones were patients with cardiac surgery [56].

Another major cause of AKI is cardiogenic shock, an ailment characterized by insufficient cardiac output causing low blood pressure with symptoms of end organ hypoperfusion such as oliguria [57]. A study performed by Van den Akker et al. mentioned that, out of 39 cardiogenic shock patients admitted in ICU, 24 developed AKI within the first 48 hours of their admission [58]. The prevalence of AKI in cardiogenic shock patients is very frequent and associated with high mortality during the first 90 days of cardiogenic shock [57]. Burns can likewise lead to AKI with a very high incidence rate of 30% and with 80% mortality. A large amount of fluids loss from a burn injury causes hypovolemia and a substantial decline in cardiac output resulting in decreased renal flow and leading to ischemia and cellular death. The ischemia further aggravates free radical formation and cellular structure damage ultimately causing additional kidney injury [59]. Drugs are notorious for causing nephrotoxicity with 20 to 40% AKI cases due to medications, this ratio reaching 60% among the elderly population. Aminoglycosides and other antimicrobials (antivirals and antifungals) are the most common drug classes responsible for AKI [60]. Other potential classes of drugs include nonsteroidal anti-inflammatory drugs (NSAID's), angiotensin-converting enzyme inhibitors (ACE), and calcineurin inhibitors. In case of appearance of any AKI symptoms in patients during therapy, drug administration should be stopped [61]. Besides, numerous infectious diseases are responsible for AKI such as malaria, leptospirosis, dengue, yellow fever, and scrub typhus. Similarly, animal venoms such as snakes and various arthropods also cause AKI [62].

#### 3.2.3. Diagnosis

The diagnosis of AKI has been evolved over the time. Conventionally, AKI diagnosis is based on the measurement of serum creatinine (SCr) and the reduction in urine yield. The definition of AKI by Acute Dialysis Quality Initiative (ADQI) group based on Risk, Injury, Failure, Loss, End-stage (RIFLE) criteria has been modified by the AKI-network (AKIN) with slight amendments [63, 64]. These two definitions and classification benchmarks of AKI were merged in 2012, and consequently, Kidney Disease Improving Global Outcomes (KDIGO) criteria came into existence. In view of the KDIGO criteria, AKI is diagnosed if SCr ≥0.3 mg/dl within 48 hours or escalating by 1.5 times from the baseline level within a week or less. AKI stages were classified by determining changes in SCr or urine output [65].

Current criteria of AKI diagnosis are widely used and accepted but have, regrettably, limitations. Both SCr and urine output are imperfect biomarkers as compared to other biomarkers which may characterize AKI in its earlier stages [66, 67]. For the subclinical diagnosis of AKI, two types of novel biomarkers known as damage and stress biomarkers are used. The novel damage biomarkers are the neutrophil gelatinase-associated lipocalin (NGAL) and the kidney injury molecule 1 (KIM-1), whereas the insulin-like growth factor binding protein 7 (IGFBP-7) and tissue inhibitor of metalloproteinase-2 (TIMP-2) belong to the novel stress biomarkers. These biomarkers may predict the detection of AKI, but their clinical application is still uncertain [68, 69]. A combo of urinary TIMP-2 and IGFBP-7 called as nephron-check has been recognized by the FDA for the diagnosis of AKI [70]. Other tools that are used for AKI diagnosis are clinical imaging techniques involving ultrasonography, contrast-enhanced ultrasonography, computerized tomography, conventional B-mode imaging, and magnetic resonance imaging [66]. AKI is a severe disease, progressive in nature, meaning that continued insult will lead to increased injury and loss of organ function with serious consequences such as death. Timely diagnosis of AKI will lead the clinician to intervene and make a proper treatment plan [71].

#### 3.2.4. Treatment of AKI Using Drugs and Plant-Based Therapies

Treatment and management plans for AKI are based on its causative agent. Those patients in whom AKI is not developed but display a risk factor or been exposed to a risk factor must undertake clinical assessment and investigation [72]. As AKI is a multifactorial and heterogeneous disease often accompanied by comorbidities, and identification of an appropriate pharmacological approach, which can assist in full cure, is quite challenging. Moreover, at the time of the diagnosis, the disease is almost established in most of the cases. AKI patients frequently suffer from increased potassium levels, metabolic acidosis, fluid overload, or increased level of blood urea due to decreased GFR. The pharmacological therapy is commonly based on treating these symptoms rather than the disease itself [73].

Various drug classes are used for the treatment of AKI according to its triggering factors. For instance, vasopressors, diuretics, as well as intravenous (I/V) fluids are administered for management of the oliguria which is related with decreased GFR in addition to an increase in salt and water retention. The purpose of this therapy is to resume a normal cardiac output, a systemic hypotension, and a neuroendocrine response. However, as observed in many studies, this therapy leads to shoddier organ function loss with worse consequences in routine surgery cases. Maintaining this therapy for a long time is also challenging and leads to many adverse responses such as interstitial edema and organ dysfunction [74].

Loop diuretics are being used based on a well-known notion that it transforms oliguric AKI patients into nonoliguric ones providing an electrolyte balance [75]. Regardless of its extensive prescription in AKI patients, improvement of the clinical picture is still missing. Moreover, some data suggest more damage compared to benefits in selected cases [76]. The results of the study carried out by Mehta et al. have shown that diuretic administration in critically ill patients suffering from kidney diseases is accompanied with increased rate of mortality and irreversible renal function loss [77]. A meta-analysis by Ho and Power has mentioned that furosemide, a loop diuretic, when used in AKI patients, has no impact on mortality ratio and risk of renal replacement therapy (RRT) reduction [78].

Among the renal vasodilators, dopamine has shown inability to protect or change the progression of ischemic AKI. Likewise, fenoldopam induces a dose-dependent hypotension which may aggravate AKI. However, according to another review based on 13 studies regarding fenoldopam role in patients enduring cardiovascular surgery, it was reported that it may decrease the RRT and in-hospital mortality rate [79]. Another pharmacological intervention in AKI is statin therapy. A meta-analysis by He et al. showed that pretreatment and posttreatment with statins in patients undergoing cardiac surgery increases the risk of cardiac surgery associated with AKI, the risk being higher with rosuvastatin compared to atorvastatin [80]. Many drugs display encouraging effects in specific stages of AKI, but none of them have shown any assured evidence in the protection of AKI. Many factors may be responsible for such a failure, e.g., most pharmacological therapies are targeting only a single pathway. Similarly, there is vagueness on initiation, discontinuation, and exact dosing for a given pharmacological therapy [81]. Obstacles in clinical trials is another accountable factor, for instance, secondary diseases in enrolled patients for clinical trials are mainly responsible for increased mortality ratio. Likewise, lack of agreement on a common definition of AKI, and its complex etiology are additional factors responsible for insufficient pharmacological options to treat this ailment [82].

With the expansion of research, new cellular and subcellular information has become available regarding the pathophysiology of AKI. As a result, more emphasis has been laid on inflammation, oxidative stress, and immune response modulation [83]. One essential condition of AKI is a kidney I/R injury, which is accompanied by inflammatory responses such as macrophage and neutrophil infiltration. Similarly, mitochondria are also affected due to ROS generation, causing changes both at cellular and vascular levels [27, 84]. Renal I/R injury leads to the reduction of antioxidant molecules such as glutathione and an increase in lipid peroxidation which can be identified by an enhanced level of malondialdehyde [84]. The generation of ROS, inflammatory molecules, and activation of apoptotic pathways and the caspase pathway leads to renal cytotoxicity and initiate a vicious circle of cell injury [85].

Another emerging pharmacological therapy is the use of plant-derived extracts and natural compounds with antioxidant and anti-inflammatory properties. Moreover, these latter ones act via multiple mechanisms to protect cell injury from ROS and inflammatory cytokines [86]. Since ancient times, many plants are used for treatment purposes and are still in practice all over the world [87, 88]. The use of medicinal plants for treatment purposes is based on hundred-year-old beliefs and innumerable experiences [89–91].

There is a growing interest in developing medicinal plant-derived products as treatments all over the world. The modern pharmaceutical industry is also capitalizing in research based on new chemical entities (NCEs) from medicinal plants. Currently, among the approved NCEs from natural sources, 25% are derived from plants. Moreover, in some therapeutic areas such as oncology, there are 60% approved plant-derived medicines [92–94]. According to the World Health Organization (WHO), 65–80% population are using plant-derived medicines in developing countries [95].

As stated previously, oxidative and inflammatory stresses are the main factors contributing to the pathophysiology of I/R injury [96–99]. In this context, easy availability and accessibility to plant-derived therapies are of prime importance. Plants represent a rich source of phytochemicals which have a high potential to act both as exogenous antioxidant and anti-inflammatory agents, as evidenced by many studies [27, 100–104]. Therefore, treating I/R-induced AKI with plant-derived extracts and compounds is the most practical approach. The aim of this review is to enlist such plant-based therapies which are tested in experimental models of renal I/R injury, thus providing an insight for future research.

Plant-based therapies provide nephroprotection against I/R-induced AKI mainly by increasing the levels of antioxidant enzymes such as superoxide dismutase (SOD) and catalase as well as glutathione levels, thereby producing antioxidant effects against ROS [105–108]. Similarly, they provide an anti-inflammatory effect mostly by inhibiting inflammatory cytokines like the tissue necrosis factor alpha (TNF-*α*), interleukin 1*β* (IL-1*β*), and interleukin-10 (IL-10) [100, 109, 110].

The nephroprotective role provided by various plants against I/R injury can be attributed to their rich contents in phytochemicals such as flavonoids, phenols, polyphenolics, alkaloids, tannins, terpenes, and saponins. Many plants contain more than one of these highly antioxidant and anti-inflammatory compounds. A summary of the various effects of plants against I/R induced-AKI is presented in Table 1.

## 4. Mechanisms by Which Plants Ameliorated Renal I/R Injury-Induced AKI

### 4.1. Increasing Antioxidant Levels

Plant-derived extracts and natural compounds ameliorated kidney I/R injury-induced AKI by increasing the levels of antioxidant enzymes and antioxidants (Table 1). These enzymes include superoxide dismutase (SOD), catalase, and glutathione peroxidase (GPx). The SOD provides natural defense against oxidative stress as it converts O_2_ into H_2_O_2_ (equation (1)). H_2_O_2_ does not contain any unpaired electrons and as such is not a free chemical radical. However, H_2_O_2_ can penetrate easily into cells and act as a poor oxidizing agent. The catalase and GPx then detoxify H_2_O_2_ into H_2_O, O_2_, and H_2_O, respectively (equation (2) and (3)) [165, 166].

(1) $$\begin{array}{c} 2{\text{O}}_{2}^{-}+2\text{H}\overset{\text{SOD}}{⟶}{\text{H}}_{2}{\text{O}}_{2}+{\text{O}}_{2}, \end{array}$$

(2) $$\begin{array}{c} 2{\text{H}}_{2}{\text{O}}_{2}\overset{\text{catalase}}{⟶}2{\text{H}}_{2}\text{O}+\frac{1}{2}{\text{O}}_{2}, \end{array}$$

(3) $$\begin{array}{c} {\text{H}}_{2}{\text{O}}_{2}\overset{\text{GPx}}{⟶}{\text{H}}_{2}\text{O}+\frac{1}{2}{\text{O}}_{2}. \end{array}$$

A number of plant-derived extracts and natural compounds (Table 1) are able to increase the levels of these antioxidant enzymes, thereby providing protection against RIRI-induced AKI. There are numerous plants, which increase the levels of SOD as shown in example (Table 1) [28, 30, 109, 110, 126]. Similarly, some plant extracts were reported to increase both SOD and catalase levels [106, 116, 121, 136, 148], while others were reported to increase all the three antioxidant enzymes SOD, catalase, and GPx [85, 105, 107, 123, 129, 146, 150, 152]. It is therefore concluded that different plants exhibited their own mechanism of protection (Table 1).

Antioxidant compounds such as glutathione (GSH) modify the cell response against ROS generation in I/R injury-induced AKI. GSH plays an important role in both the detoxification of drug metabolites and the regulation of gene expression and apoptosis [167]. Depletion of GSH levels lead to an increase in oxidative stress and is directly associated with I/R injury [168]. A number of plants as shown in Table 1 were reported to increase the levels of GSH, thereby protecting kidney from ROS generated by I/R injury [27, 111, 118, 120, 122] (Table 1), and were reported to increase the levels of GSH, SOD, and catalase [116, 145], while some others have been found to increase GSH, GSH-Px, SOD, and catalase [152]. Thus, plants exhibited versatile mechanisms of protection.

### 4.2. Decreasing Anti-Inflammatory Cytokines

The dying and injured cells as a consequence of renal I/R injury release proinflammatory cytokines such as interleukins and tumor necrosis factor (TNF) and chemotactic cytokines (CCL5, CCL2, and CXCL2). There is also an activation of some transcription factors such as heat shock proteins (HSP), high mobility group box-1 (HMGB1), and hypoxia-inducible factor-1 (HIF1) [169, 170]. These factors are responsible for the stimulation of cell surface receptors, which in turn triggers inflammatory and cytotoxic reactions [171].

Plant-derived extracts and natural compounds target proinflammatory cytokines to halt inflammation and ameliorate renal I/R injury-induced AKI. The main inflammatory cytokines and chemokines which are decreased by plant-derived extracts include tissue necrosis factor alpha (TNF-*α*), interleukin-1*β* (IL-1*β*), interleukin-10 (IL-10), interleukin-6 (IL-6), interleukin-8 (IL-8), and interferon gamma (IFN-*γ*) [172]. Natural compounds that decrease the levels of TNF-*α*, IL-1*β*, and IL-6 include arctigenin [117], luteolin [106], epigallocatechin gallate [125], pycnogenol [154], and ferulic acid [132] as well as plant-derived extracts such as *Juglans mollis* [109] and *Sonchus oleraceus* [110] (Table 1).

**Figure 1:**
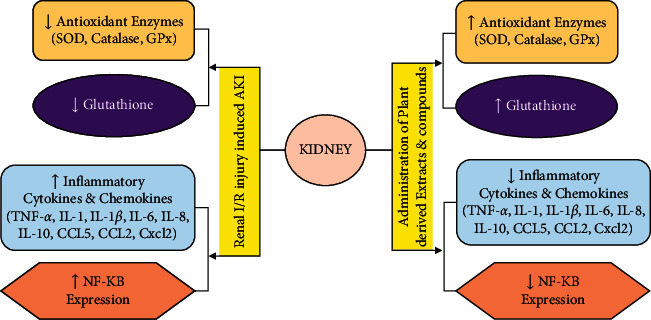
Effect of renal ischemic reperfusion (I/R) induced acute kidney injury (AKI) and administration of plant-derived extracts and compounds on kidney. The upward arrow shows “increase,” and downward arrow direction shows “decrease.”

The TNF-*α* induces proinflammatory effects by activating transmembrane TNF-*α* receptors, causing the stimulation of the nuclear factor-*κ*B (NF-*κ*B). NF-*κ*B is responsible for the expression of over 400 genes including cyclooxygenase-2, lipoxygenase-2 (LOX-2), inducible nitric oxide synthase (iNOS), and the transcription of inflammatory cytokines and chemokines [173, 174]. The peak levels of NF-*κ*B in rat models of renal I/R injury AKI were found after 15 minutes of reperfusion [173]. Therefore, inhibiting the NF-*κ*B-mediated inflammatory pathway is another approach to ameliorate I/R injury induced-AKI inflammation [175]. Many plant-derived compounds were found to inhibit or decrease the levels of NF-*κ*B expression such as ursolic acid [164], epigallocatechin gallate [125], aloperine [113], arctigenin [117], and cannabidiol [122] (Table 1). The effects of renal ischemic reperfusion (I/R) induced acute kidney injury (AKI) and administration of plant-derived extracts and compounds on kidney have been illustrated in Figure 1.

### 4.3. Increasing Adenosine Levels

Adenosine is an endogenous nucleotide composed of adenine and ribose. Adenosine plays an important role against hypoxia as a result of renal I/R injury [176, 177]. Adenosine regulates essential kidney functions such as the glomerular filtration rate (GFR), renin release, and tubular glomerular feedback mechanisms [178]. Adenosine produces its effects on kidney via the stimulation of adenosine receptors (AR), which has four subtypes (A1AR, A2AAR, A2BAR, and A3AR). Necrosis, apoptosis, and inflammation due to renal I/R injury induced-AKI are reduced by adenosine via stimulation of the A1AR receptor [179]. Adenosine has 100 times more affinity to bind with A1AR and A2AR receptors compared to the other two subtypes of AR receptors. Stimulation of both A1AR and A2AR receptors play a role in controlling inflammation after renal I/R injury-induced AKI [180].

Various plant-derived extracts and compounds (Table 1) were reported to increase the levels of adenosine activity. Pretreatment with sesamin [161], mangiferin [141], and ferulic acid [132] remarkedly increased adenosine levels in the I/R treatment group compared to the renal I/R injury AKI group. Increases in adenosine levels were accompanied by a decrease in caspase-3 expression and inflammatory cytokine levels, which shows a reduction in apoptosis, necrosis, and inflammation, respectively.

### 4.4. Other Mechanisms

The phosphatidylinositol-3-kinase (PI3K)/Akt/mTOR pathways play a significant role in cell survival processes such as apoptosis, metabolism, and angiogenesis. These pathways are so interrelated that, in many circumstances, they are regarded as a single pathway [181, 182]. The PI3K/Akt/mTOR signaling is assumed to be involved in renal I/R injury-induced inflammation [183, 184]. Among the compounds listed in Table 1, only aloperine was found to regulate the PI3K/Akt/mTOR pathway and have markedly reduced its signaling compared to renal I/R injury AKI group [113].

Another pathway known as Janus kinase/signal transducers and activators of transcription (JAK/STAT) is essential for growth hormone and other cytokine signaling. The cytokines bind to their receptors and activates JAK, which then causes the phosphorylation of STAT. The STATs are then transferred into the nucleus where they initiate target gene expression [185, 186]. The JAK/STAT pathway is supposed to be involved in renal I/R injury [187]. Among the JAK/STAT pathways, the subtype JAK2 signaling via STAT1 and STAT3 is the best studied in diseases affecting kidney [188]. Among the natural compounds enlisted in Table 1, apigenin [114] and ursolic acid [164] have been found to ameliorate renal I/R injury induced-AKI via action on the JAK2/STAT3 pathway.

The sonic Hedgehog (Shh) is a glycoprotein and a key ligand of the Hedgehog pathway. It has an important role in cell differentiation and apoptosis and governs the embryonic development. The Shh signaling has diffused effects on various organ systems [189, 190]. The Shh signaling is also known to have a role in kidney development and tissue repair after injury. Its release is often induced in postischemia of a tissue and regulates important biological processes, for instance, antiapoptosis and antioxidant effects, thereby promoting tissue repairing [191, 192]. Polydatin (Table 1) ameliorates renal I/R injury induced-AKI by activating the Shh signaling pathway [107]. This latter study suggests that the Shh pathway can constitute a new target to treat renal I/R injury induced-AKI in the future.

Heat shock proteins (HSPs) regulate normal cell functions in kidney after I/R injury. Among HSPs, HSP70 is the most studied molecule as it has cytoprotective characteristics and is often chosen as a therapeutic target. The HSP70 is known to be involved in anti-inflammatory and antiapoptotic effects and in the stimulation of regulatory T-cells in renal I/R injury [193]. Among the plant-derived compounds (Table 1), dioscin has been found to ameliorate renal I/R injury-induced AKI via the upregulation of HSP70. Upregulation of HSP70 results in the inhibition of TLR4/MyD88 signaling and cyclooxygenase-2 (COX-2) pathways [124].

## 5. Effects on Biomarkers and Renal Tissues

Renal I/R injury or other diseases such as hypertension, genetic disorders, infections or toxins leads to AKI, which ultimately causes a decrease of the renal function. Biomarkers of the renal function are assessed to evaluate the severity of kidney injury, identify risk factors, and more importantly to analyze responses towards the applied therapies [194]. Serum creatinine (SCr) is still the gold standard biomarkers to diagnose the AKI according to recent KDIGO classification system [65]. The majority of the plant-derived extracts and compounds listed in Table 1 decrease the levels of SCr and blood urea nitrogen (BUN). However, there is accumulative evidence in the literature which shows that SCr and BUN have many limitations and are therefore considered as suboptimal biomarkers [195–197]. Cystatin C, a renal biomarker, has many advantages over creatinine. It is a small size protein (13 kDa) and is generated by all nucleated cells, as compared to SCr which is reliant on muscle mass [198]. For patients displaying muscular complications, cystatin C offers a clinical edge over creatinine [199]. Cystatin C has also limitations as its level increases in conditions like hyperthyroidism, during corticosteroid use and when there is a high cell turnover [200]. Plant extracts or compounds showing an effect on cystatin C levels include *Nigella sativa* oil [146] and curcumin [123] (Table 1). The biomarkers NGAL and KIM-1 are considered to diagnose AKI in its early stages compared to other surrogate biomarkers [201, 202]. Among the plant-derived extracts and compounds which lowered NGAL and KIM-1 levels are quercetin [155], lycopene [140] and *α*-bisabolol [29], oleanolic acid [85], as well as acai fruit extracts [112], respectively (Table 1).

The renal I/R injury induced-AKI causes necrosis, apoptosis, hemorrhages, vascular congestion, inflammatory cell infiltration, cellular edema, and other degenerative changes in kidney tissues [27, 104, 131, 148]. Plant-derived extracts and compounds have shown marked effects on kidney tissues, almost all the plants effectively lowered the total renal histopathological scores (Table 1).

## 6. Discussion

Plants displaying a nephroprotective capability against I/R-induced-AKI have been summarized (Table 1). According to Amin and Khan (2016), natural products represent an incredible source for the development of new molecules for drug discovery and development. Moreover, since the last three decades, 35% of the newly developed molecules are derived from natural sources. Plants having therapeutic potential are being used for numerous pathological conditions for many centuries [203, 204].

I/R injury is considered as one of the major clinical problems for clinicians, specifically during hospital surgeries often leading to loss of function in tubular epithelial cells and causing AKI accompanied by other complications. AKI induced by I/R injury includes oxidative stress in addition to activation of immune responses and upregulation of cytokines and chemokines [205]. Kidneys are very vulnerable to the I/R effects, progressive injury, and unmet oxygen requirements resulting in dehydration as well as electrolytic imbalance with increased morbidity and mortality [14]. Renal I/R injury induced-AKI causes a series of biochemical and pathophysiological changes that are reflected as alterations in the levels of biomarkers used clinically for the diagnosis and the monitoring of the general health status of the patients. Research involving the use of pharmacologically active molecules in the prevention and treatment of ischemic pathologies uses biomarkers as assessment tools. However, it is important to recognize their applications, limitations, and their proper interpretation before using them [206].

Almost all the plants included in this review possess activity against I/R-induced-AKI by reducing serum creatinine in addition to blood urea nitrogen (BUN), while many others decrease the cystatin C levels, for instance, garlic oil, *Nigella sativa* oil, and curcumin (Table 1). Similarly, NGAL and KIM-1 are lowered by quercetin, lycopene and *α*-bisabolol, oleanolic acid, as well as acai fruit extracts, respectively (Table 1). The RIRI-induced AKI causes severe renal histopathological changes. The major renal histopathological changes that were observed in most of the studies (Table 1) were cell vacuolization, interstitial hemorrhage foci, glomerular congestion, inflammatory infiltrate, cellular damage in loop of Henle, moderate to severe necrosis, hyaline cast, and loss of brush border [26, 118, 119, 133, 135]. In agreement with the biochemical results, almost all the plants effectively lowered the total renal histopathological score as well. The oral route is the most desired route of drug administration because it has many benefits, e.g., ease of administration, patient compliance, and flexibility in dosage form. Majority of the plant extracts and compounds (Table 1) were administered via oral route. Moreover, most of the active pharmaceutical products are being used orally. All these plant extracts and derivative compounds have the potential to be developed as a therapeutic entity that can be administered orally for the treatment of RIRI-induced AKI.

The plant-derived extracts and compounds (Table 1) ameliorate renal I/R injury via different mechanisms, but the antioxidant and anti-inflammatory mechanisms were found as the most prominent ones. The oxidative stress levels were reduced mostly via increasing the levels of the endogenous antioxidants such as the total flavonoids from *Rosa lavigata* Michx fruit, which reduced in vivo oxidative stress by increasing SOD, GSH, and GSH-Px and decreasing MDA levels. Similar effects have been observed with caffeic acid which also increases catalase levels. Moreover, polysaccharides in *Lycium barbarum*, the bark of *Juglans mollis*, luteolin, ethanolic extracts of *Hypericum perforatum*, apigenin, oleanolic acid, berberine, and many others, as listed in Table 1, are some of the examples of plant-derived extracts and compounds having an effect against one or more of these oxidative stress parameters.

Because of the redox imbalance, a local and systemic inflammatory process is activated. Renal I/R injury initiates several inflammatory reactions within the renal parenchyma. Besides infiltration of neutrophils, renal I/R injury is accountable for the generation of many inflammatory cytokines, for instance, IL-6, IL-1, and TNF-*α* (Thurman, 2007). An anti-inflammatory activity was displayed by various plant extracts (Table 1) against inflammatory cytokines, e.g., the ethanolic extracts of *Sonchus oleraceus* decreased the IL-6, IL-1*β*, and TNF-*α* levels compared to I/R groups. Similar effects were also observed with leaf and bark extracts of *Crateva nurvala*, ferulic acid, arctigenin, luteolin, seasamin, mangiferin, epigallocatechin gallate, and ethanolic extracts of *Salvia miltiorrhiza*, while others display activity against one or more of these inflammatory parameters (Table 1).

This review demonstrated that I/R process causes a series of intracellular events altering homeostasis and renal function. These events are related to the production and the accumulation of ROS, which cause oxidative stress, thus altering important functions of energy metabolism such as mitochondrial transmembrane potential. These phenomena interrupt oxidative phosphorylation, inhibiting the production of ATP, and causing energy deficit and consequent cell death. This can lead to tissue damage that, if not reversed quickly, can cause chronic disease [111]. Natural antioxidant substances are shown to be nephroprotective and the mechanisms of their action are well described. The experimental data endorse the role of antioxidant therapy as observed from the studies summarized in Table 1. Further toxicological, pharmacological, and human studies are warranted to develop these entities into lead drugs or final therapeutic molecules.

## 7. Conclusion

Renal I/R injury is the leading cause of AKI, which is associated with high ratios of mortality and morbidity. Renal I/R injury is responsible for the generation of ROS and RNS leading to increase in oxidative and inflammatory stress. These stresses induce proinflammatory cytokines and depletion of antioxidant enzymes as well as antioxidant compounds, activating several pathways and leading to cell necrosis and apoptosis. Recently, several clinical biomarkers and molecular targets of AKI have been discovered. However, there is no defined biomarkers or targets, which can be used for the treatment of AKI. Various natural plant-derived therapies have shown best outcomes in animal studies. These plant-derived extracts and compounds have only been tested in selected transgenic animal models. To develop them into an efficacious therapeutic entity, they should be further tested in proper vertebrate multitransgenic animal models so that their human use can be validated. Many plant-derived extracts and compounds presented in this study have shown nephroprotective effects against renal I/R injury induced-AKI via elevating antioxidant activities, decreasing inflammatory cytokines, and increasing adenosine levels and activating other pathways. Future research should be focused on toxicity, pharmacokinetic profiling, and effective dose concentration for the design of clinical trials for the treatment of renal I/R induced-AKI.

## Figures and Tables

**Table 1:** Plant-derived extracts and compounds having nephroprotective effects against renal ischemic reperfusion injury-induced acute kidney injury.

Substance used	Source	Experimental model	Ischemia	Mechanism	Route of Administration and order	Effects	Reference
(−)-*α*-Bisabolol	*Matricaria chamomilla, M. crassifolium, Salvia runcinata*, *Vanillosmopsis* sp.	Male Wistar albino rat (in vivo) LLC-MK 2 cells (in vitro)	Right renal nephrectomy and left kidney ischemia for 60 min followed by 48 h reperfusion	Antioxidant, anti-inflammatory, antiapoptotic	Oral route posttreatment	↓Urine osmolality ↓SCr, urea, uric acid, MDA ↓FENa^+^, FEK^+^, FECl^−^ ↓uKim-1, ↓Proteinuria, Albuminuria ↓Renal histopathological score ↑Creatinine clearance, GSH ↑Water consumption, diuresis ↑Cell viability	[26]
	Various plants and herbs, e.g., in essential oil of *Matricaria chamomilla*	HK2 cells	In vitro ischemic reperfusion model by the anaerobic chamber method. Firstly, ischemia was induced for 24 h followed by 3 h reperfusion.	Antioxidant, antiapoptotic	Posttreatment	↓Cell apoptosis ↓TBARS ↓KIM-1 ↑Cell viability ↑GSH Inhibit NADPH oxidase 4	[111]

Acai fruit extract	*Euterpe oleracea*	Male Wistar albino rat	Bilateral ischemia for 45 min followed by 6 h reperfusion	Antioxidant, antiinflammatory	Oral route pretreatment	↓SCr, BUN, renal KIM-1 ↓MDA, MPO, IFN-*γ*, caspase-3 ↓Collagen IV, endothelin-1 ↑IL-10	[112]
Aloperine	S*ophora alopecuroides*	C57BL/6 mice RAW264.7 and HK2 cells	Bilateral ischemia for 45 min followed by 24 h reperfusion	By regulating PI3K/Akt/mTOR signaling and NF-*κ*B transcriptional activity		↓IFN-ϒ, IL-1*β*, caspase-3 ↓NF-*κ*B expression (*in-vitro & in-vivo*) ↓PI3K/AkT/mTOR pathway signaling ↑IL-10, SOD	[113]

Apigenin	Celery parsley wheat sprouts	Male Sprague Dawley rat NRK-52E cells	Bilateral ischemia for 45 min followed by 24 h reperfusion	Activation of the JAK2/STAT3 pathway	Intraperitoneal route pretreatment	↓SCr, BUN, MDA ↑SOD and GPx levels in vivo and in vitro ↑JAK2 and STAT3 phosphorylation in vivo and in vitro ↑Bcl-2 and procaspase-3 expression ↓Bax and caspase-3 expression	[114]

Aqueous extract	*Cuscuta chinensis* (seeds)	Male Sprague Dawley rat	Bilateral ischemia for 45 min followed by 4 days reperfusion	By upregulation of water channels and Na^+^K^+^ ATPase	Oral route Posttreatment	↓Urine volume ↑Urine osmolality ↑Urinary Na^+^, K^+^, Cl^−^ ↑Creatinine clearance ↑AQP-2, AQP-3 expression ↑Na^+^K^+^ ATPase	[115]

Aqueous extract	*Murraya koenigii* (leaves)	Male Wistar albino rat	Unilateral ischemia in the left kidney for 60 minutes followed by reperfusion	Antioxidant	Oral route pretreatment and posttreatment	↓SCr, BUN, MDA, MPO ↓Proteinuria ↑SOD, CAT, GSH	[116]

Arctigenin	*Arctium lappa* (fruit)	Male C57BL/6 mice	Bilateral ischemia for 30 min followed by 24 h reperfusion	Anti-inflammatory effect	Oral route pretreatment	↓IL-1*β*, IL-6, TNF-*α* ↓IL-10 ↓TLR4/Myd88 protein expression ↓NF-*κ*B expression	[117]

Ascorbic acid	Potatoes, green leafy vegetables, root vegetables, citrus fruits etc.	Male Sprague Dawley rat	Right nephrectomy with left renal ischemia for 45 min followed by 3 h reperfusion	Inhibiting oxidative stress	Intraperitoneal route pretreatment	↓SCr, BUN, LDH, MDA ↓Renal histopathological score ↑GSH	[118, 119]
Berberine	*Berberis vulgaris*, *Hydrastis canadensis*, *Coptis chinensis*, *Arcangelisia flava*, *B. aquifolium*, *B. aristata*	Male Wistar albino rat	Right renal nephrectomy and left kidney ischemia for 45 min followed by 4 weeks reperfusion	Nephroprotection by caspase mitochondria dependent pathway	Oral route posttreatment	↓SCr, BUN, MDA, MPO ↓Na-K-ATPase and Ca-ATPase ↓KIM-1 and TNF-*α* mRNA expression ↓Bax, ↓Caspase-3 mRNA expression ↑Renal SOD and GSH ↑Bcl2 mRNA expression	[30]
Betulinic acid	*Betula alba* (bark)		Right nephrectomy with left renal ischemia for 45 min followed by 6 h reperfusion	Inhibit leukocyte apoptosis and upregulation of Na^+^K^+^ ATPase	Intraperitoneal route pretreatment	↓SCr, BUN, LDH, MDA ↓TNF-*α*, MPO ↓Leukocyte apoptosis ↑Na^+^K^+^ ATPase, GSH	[120]
Caffeic acid	Commonly found in grains, fruits, and dietary add-ons as simple esters with quinic acid or saccharides		Bilateral ischemia reperfusion for 90 minutes followed by reperfusion for 24 hours	Inhibit 5-lipoxygenase pathway. Anti-inflammatory and antioxidant effect	Oral route pretreatment	↓SCr, BUN, MDA, ↓TNF *α* ↑Catalase, SOD	[121]

Cannabidiol	*Cannabis sativa*	Male Sprague Dawley rat	Bilateral ischemia for 30 min followed by 24 h reperfusion	Antioxidant, anti-inflammatory, antiapoptotic activity	Intravenous pre- and posttreatment	↓SC, MDA, NO ↓iNOS, TNF-*α*, COX-2 ↓ NF-*κ*B, FasL caspase-3 ↑GSH	[122]

Curcumin	*Curcuma longa*	Male Wistar albino rat	Bilateral ischemia for 45 min followed by 24 h days reperfusion	Antioxidant, free radical scavenging	Oral route pretreatment	↓SCr, BUN, cystatin C ↓MDA, NO ↓Total oxidant status ↑SOD, GPx (serum and renal) ↑Catalase (renal) ↑Total antioxidant capacity	[123]
Dioscin	*Dioscorea nipponica*	Male Sprague Dawley rat NRK-52E and the HK-2 cells	Bilateral kidney ischemia for 45 min followed by 24 h reperfusion	Inhibiting theTLR4/MyD88 signaling pathway via upregulation of HSP70		↓SCr, BUN ↓TLR4, MyD88, TRAF6 in vitro and in vivo ↓IL-1, IL-6, TNF-*α*, ICAM-1 ↓IFN-ϒ in vitro and in vivo ↑HSP 70 in vitro and in vivo ↑Cell viability	[124]

Epigallocatechin gallate	*Camellia sinensis*	Male Sprague Dawley rat	Right nephrectomy and left kidney ischemia for 45 min followed by 24 h reperfusion	Anti-inflammatory suppressing NF-*κ*B decreasing apoptosis	Intraperitoneal route pretreatment	↓SCr, BUN ↓TNF-*α*, IL-6, IL-1*β* ↓Cleavage caspase-3 and Bax ↑Caspase-3 and BCL-2 Suppressing NF-*κ*B	[125]
Ethanolic extract	*Apium graveolens* (leaves and stem)		Bilateral ischemia for 45 min followed by reperfusion	Antioxidant and anti-inflammatory	Oral route pretreatment and posttreatment	↓SCr, urea ↑SOD and nitrogen oxide level	[126]
	*Crateva nurvala* (leaves and bark)	Male Wistar albino rat	Unilateral ischemia for 1 hour followed by 24 h reperfusion	Antioxidant and anti-inflammatory	Oral route pretreatment	↓SCr, BUN, LDH, MDA ↑NR-F2, GSH ↓TNF-*α*, IL-6, caspase-3	[27]
	*Sonchus oleraceus*		Bilateral ischemia for 45 min followed by 15 h reperfusion	Antioxidant and anti-inflammatory	Pretreatment	↓SCr, BUN, MDA ↑SOD ↓IL-6, IL-1*β*, TNF-*α*	[110]
	*Brassica rapa* (roots)		Bilateral ischemia for 60 min followed by 24 h reperfusion	Anti-inflammatory antioxidant		↓SCr, urea, uric acid ↓Renal tissue hemorrhage ↓Necrosis and tubular distention	[127]
	*Hypericum perforatum* (flowering herb)	Male Sprague Dawley rat	Left renal ischemia for 45 min with right renal nephrectomy followed by 3 h reperfusion	Antioxidant and anti-inflammatory	Intraperitoneal route pretreatment	↓SCr, BUN, MDA ↑SOD, catalase, GPx	[105]
	*Petroselinum cripum*	Male Wistar albino rat	Bilateral renal ischemia for 30 min followed by reperfusion for 24 hours	Attenuating oxidative stress and inflammation		↓SCr, BUN, MDA, ↓NO, ICAM-1, TNF-*α* ↓Leukocyte infiltration rate ↑Glomerulus dimeter, FRAP level	[128]
	*Salvia miltiorrhiza*		Bilateral ischemia for 60 min followed by 30 min reperfusion	Antioxidant anti-inflammatory	Oral route pretreatment	↓SCr, BUN, MDA ↓IL-6, IL-8, TNF-*α* ↑GSH, SOD, catalase, GPx	[129]
	*Tribulus terrestris*	Male Sprague Dawley rat	Bilateral ischemia for 30 min followed by 24 h reperfusion	Antioxidant		↓SCr, BUN, MDA ↓Na^+^, K^+^ excretion, FRAP ↑Urine osmolality, ↑Creatinine clearance	[130]
	*Dalbergia ecastaphyllum*	Male Wistar albino rats	Right nephrectomy and left kidney ischemia for 60 min followed by 48 h reperfusion	By reducing oxidative stress, eNOS, and heme-oxygenase upregulation		↓SCr, serum urea ↓Renal and urine MDA, GSH Restores eNOS expression ↑Heme-oxygenase expression	[131]

Ferulic acid	Commelinid plants, grasses, grains, vegetables, flowers, fruits, leaves, beans, seeds of coffee, artichoke, peanut, and nuts	Male C57/BL6 mice	Bilateral ischemia for 35 min followed by 24 h reperfusion	Increasing adenosine generation via HIF-1*α*	Oral route pretreatment	↓SCr, BUN, caspase-3 ↓IL-1*β*, TNF-*α*, MPO activity ↑Adnesine activity ↑CD 73, CD 39 ↑HIF-1*α* mRNA	[132]
Garlic juice	*Allium sativum* (bulbs)	Male Wistar albino rats	Right nephrectomy with left renal ischemia for 45 min followed by 24 h reperfusion	Antioxidant, antiapoptotic		↓SC, urea, FENa^+,^ FEK^+^ ↓Renal histopathological score ↑Urinary creatinine	[133]
Garlic oil			Bilateral ischemia for 45 min followed by 6 h days reperfusion	Antioxidant, anti-inflammatory		↓SC, Urea, Cystatin C ↓Oxidative stress index ↓MPO, NO	[134]
Ginger	*Zingiber officinale*		Right nephrectomy with left renal ischemia for 45 min followed by 24 h reperfusion	Antioxidant, anti-inflammatory		↓SC, urea, FENa^+^ ↑Urinary creatinine and urea ↑Urinary K^+^ excretion ↓Renal histopathological score	[135]
*Ginkgo biloba* EGb761 extract	*Ginkgo biloba*		Unilateral ischemia in left kidney for 60 minutes followed by 60 minutes of reperfusion	Antioxidant		↓MDA ↓Necrosis and cast formation ↑SOD, CAT	[136]
Hydroalcoholic extract	*Rosa canina* (fruits)		Bilateral ischemia for 45 min followed by 24 h reperfusion	Antioxidant, anti-inflammatory		↓SCr, BUN ↓Renal tissue histopathological score	[137]
	*Crocus sativus*		Bilateral ischemia for 30 min followed by 24 h reperfusion	Antioxidant and anti-inflammatory		↓SCr, BUN, MDA ↓Lymphocytes infiltration ↓TNF-*α*, ICAM-1 mRNA expression ↑FRAP	[138]
	*Juglans mollis* (bark)		Bilateral renal ischemia for 45 min followed by 15-hour reperfusion	Antioxidant and anti-inflammatory.		↓SCr, BUN, MDA ↑SOD ↓TNF-*α*, IL-1*β*, IL-6	[109]
Lavender oil	*Lavandula angustifolia*		Right nephrectomy followed by renal ischemia for 45 min followed by reperfusion for 24 hours	Attenuating oxidative stress and inflammation	Intraperitoneal posttreatment	↓SCr, BUN, MDA ↓TNF*α*, IL1*β*, IL10 ↓Apoptotic cells ↓Total histopathological score ↑SOD, GPx, catalase	[100]

Liposomes containing curcumin	*Curcuma longa*	Male C57BL/6 mice	Bilateral ischemia for 30 min followed by 24 h reperfusion	Targeted cellular delivery to renal tubular epithelial cells and antigen presenting cells conferring protection from IR injury, mediated by NF-kB	Intravenous pretreatment	Suppress NF-*к*B activity ↓SC, urea, ↓Renal histopathological score ↓TNF-*α*, CCL5, CCL2, CXCL2 ↓iNOS ↑SOD	[139]

Luteolin	Carrots, peppers, celery, olive oil, peppermint, thyme, rosemary, and oregano	Male Swiss albino mice	Bilateral ischemia	Antioxidant and anti-inflammatory	Pretreatment	↓SCr, BUN, MDA ↑SOD, CAT, GSH ↓TNF-*α*, IL-1*β*, IL-6 ↓Bax and caspase-3 expression ↑Bcl-2 expression	[106]
Lycopene	Tomato, apricots, papaya, pink grapefruit, guava, and watermelon		Bilateral renal ischemia for 30 min followed by reperfusion for 2 hours	Antioxidant and anti-inflammatory	Intraperitoneal pretreatment	↓ Scr, blood urea, plasma NGAL ↓Tissue Bax concentration ↓F21sop, ↓Notch2/HeS1 ↓Renal TLR 2, renal IL-6	[140]

Mangiferin	*Mangifera indica* also present in 16 other plant families including Anacardiacae, Gentianaceae, and Iridaceae	Male C57/BL6-mice	Left nephrectomy and right kidney ischemia for 30 min followed by 24 h reperfusion	Anti-inflammation and inducing adenosine production	Oral route pretreatment	↓SCr, BUN, ↓Plasma potassium (K^+^) ↓ Caspase-3 mRNA expression ↓TNF-*α*, IL-1*β* ↓NO, MPO ↑Adenosine and CD73 expression	[141]

Methanolic extract	*Aruncus dioicus* (whole plant)	Male Sprague Dawley rat	Right nephrectomy with left renal ischemia for 40 min followed by 24 h reperfusion	Antioxidant, antiapoptotic	Intraperitoneal route pretreatment	↓SC ↓Renal tissue histopathological score ↓Bcl-2/Bax ratio	[142]
	*Cassia mimosoides* var. *Nomame*		Right nephrectomy with left renal ischemia for 40 min followed by 24 h reperfusion	Antioxidant		↓SC ↓Caspase-3 ↓Bcl-2/Bax ratio	[143]
	*Stevia rebaudiana*		Left renal ischemia for 45 min with right renal nephrectomy	Antioxidant and anti-inflammatory	Oral pretreatment	↓SCr, FENa^+^_,_ Creatinine clearance ↓MDA ↑GSH, Catalase	[144]
	*Benincasa cerifera* (fruits)	Female Wistar albino rat	Bilateral ischemia for 60 min followed by 6 h reperfusion	Free radical scavenging activity		↓SCr, BUN, uric acid, MDA ↑SOD, catalase, GSH	[145]
*Nigella sativa* oil	*Nigella sativa* (seeds)	Male Wistar albino rat	Bilateral ischemia for 60 min followed by 24 h reperfusion	Antioxidant, free radical scavenging.		↓SCr, BUN, cystatin c. ↓MDA, NO ↓Renal histopathological score ↓Total oxidant status ↑SOD, GPx (serum and renal) ↑Catalase (renal) ↑Total antioxidant capacity	[146]
Oleanolic acid	*Olea europaea*, *Viscum album*, *Aralia chinensis*, >120 other plant species		Bilateral renal ischemia for 45 min followed by 6 h reperfusion	Antioxidant, anti-inflammatory reductions in Nrf-2	Intraperitoneal route pretreatment	↓SCr, BUN, renal KIM-1 ↓LDH, MDA, caspase-3 expression ↓IFN-*γ*, IL-6, MPO ↑SOD, GST, GPx and CAT ↑IL-10 ↑Nrf-2	[85]
Osajin	*Maclura pomifera*		Unilateral ischemia in left renal artery for 60 min followed by 10 min reperfusion	Antioxidant	Oral route pretreatment	↑SOD, GSH-Px (serum) ↑Total antioxidant capacity ↓Renal histopathological score effectively at a dose of 10 mg	[147]
Osthole	*Cnidium monnieri, Angelica pubescens, Peucedanum ostruthium*		Right nephrectomy and unilateral ischemia in left kidney for 45 min followed by 1, 6, and 24 h reperfusion	Antioxidant, antiapoptotic		↓SCr, BUN ↓Caspase-3 ↑SOD, CAT ↑Bcl-2/Bax ratio	[148]
Picroliv	*Picrorhiza kurrooa* (roots and rhizome)	Male Sprague Dawley rat	Unilateral ischemia in left renal artery for 60 min followed by 5, 60, 120, and 240 min reperfusion	Antioxidant, antiapoptotic		↓MDA (maximal at 120 min) ↑GSH (maximal at 240 min) ↑GPx, SOD ↓NO ↓Renal ICAM-1 ↓Apoptosis	[149]
Piperine	*Piper nigrum* (seeds)	Male Wistar albino rat	Bilateral renal ischemia for 30 min followed by reperfusion for 24 hours	Attenuating oxidative stress and inflammation		↓SCr, BUN, MDA ↓TNF-a and ICAM-1 mRNA expression ↓Total histopathological score ↑Total FRAP level	[104]
Polysaccharide extract	*Dipsacus asperoides* (roots)		Bilateral ischemia for 45 min followed by 24 h reperfusion	Antioxidant		↓SCr, BUN, LDH, MDA ↑SOD, CAT, GPx	[150]

Polydatin	*Polygonum cuspidatum* (roots)	Male BALB/c mice primary renal tubular epithelial cells (RTECs)	Unilateral ischemia in left kidney for 30 min followed by reperfusion	Antioxidative stress and anti-inflammation by activating the sonic Hedgehog (SHH) signaling pathway	Intraperitoneal route pre and posttreatment	↓Caspase-3 expression ↑Shh mRNA expression (in vivo and in vitro) ↑SOD, GST, GPx, and CAT (in vivo)	[107]

Polyphenols	*Camellia sinensis*	White male rabbit	Bilateral ischemia for 30, 60, 90, and 120 min, followed by 24 h reperfusion	Antioxidant, antinecrotic	Intravenous route pretreatment	↓SCr, BUN ↓Renal histopathological score ↓Immune peroxidase labeling of CD8^+^T cells in kidney tissues All the results were found significant at 90 min of ischemia	[151]

Polysaccharide peptide	*Ganoderma lucidum* (fruits)	Male C57BL/6J mice NRK-52E cells	Left nephrectomy and right kidney ischemia for 35 min followed by 24 h reperfusion	Counteracting oxidative stress	Intraperitoneal route pretreatment	↓SCr, BUN ↓MPO, MDA ↓Bax/Bcl-2 ratio, ↑SOD, CAT, GSH and GPx ↑Cell viability	[152]

Polysaccharides	*Lycium barbarum*	Wistar albino rat	Bilateral renal ischemia for 45 min followed by 24 h reperfusion	Antioxidant and anti-inflammatory inhibiting apoptosis	Oral route pretreatment	↓SCr, Serum urea, MDA ↑SOD, ↓Serum IL-1*β*, TNF-*α* Enhanced renal expression of Bcl-2 mRNA	[28]
Proanthocyanidin	Grape seed	Male Sprague Dawley rat	Bilateral ischemia for 60 min followed by 6 h reperfusion	Decreasing oxidative and nitrosative stress		↓SCr, BUN, AST ↓MDA, NOx ↓Renal histopathological score ↑SOD, GPx	[153]

Pycnogenol	*Pinus maritima* (fresh bark)	Male Wistar albino rat	Right nephrectomy with left renal ischemia for 45 min followed by 3 h reperfusion	Antioxidant, anti-inflammatory; inhibit neutrophil infiltration	Intraperitoneal route pretreatment	↓SCr, BUN, MDA ↓TNF-a, IL-1*β*, and IL-6 ↓MPO ↓Renal histopathological score ↑GSH, Na^+^K^+^ ATPase	[154]
Quercetin	Apples, berries, *Brassica* vegetables, capers, grapes, onions, shallots, tea, tomatoes seeds, nuts, flowers, barks, and leaves	Male Swiss albino mice	Bilateral renal ischemia for 30 min followed by reperfusion for 24 hours	Attenuating oxidative stress and inflammation		↓Blood urea, SCr, plasma NGAL ↑BCl-2 level ↓Tissue Bax concentration ↓F2-isoprostane ↓Renal notch-1 jagged-1 level	[155]

Resveratrol	Grapes, wine, peanuts, soy	Male Wistar albino rat	Bilateral ischemia for 40 min followed by 24 h reperfusion	Antioxidant, free radical scavenging	Intravenous route pretreatment	↓Mortality rate ↓SCr ↓TBARS ↓Renal histopathological score ↑NO	[156, 157]
Rhizome extract	*Coptidis japonica* (rhizome)		Bilateral ischemia for 60 min followed by 6 and 24 h reperfusion	Antioxidant	Oral route pretreatment	↓SCr, BUN, MDA ↑SOD, catalase ↑GSH (renal) ↓DNA fragmentation rate	[17]

Rosmarinic acid	*Rosmarinus officinalis*, *Melissa officinalis*	Male Sprague Dawley rat	Right nephrectomy and ischemia in the left kidney for 60 min followed by 60 min reperfusion	By decreasing oxidative stress	Intraperitoneal route pretreatment	↓MDA, MPO ↑SOD, GPx	[158, 159]
Rutin	Buckwheat and many vegetables, fruits, beverages such as tea and wine	Male Wistar albino rat	Right nephrectomy with left renal ischemia for 45 min followed by 3 h reperfusion	Decreasing oxidative stress, anti-inflammatory		↓SCr, BUN, LDH, MDA ↓Renal histopathological score ↑GSH, MnSOD	[160]

Sesamin	*Sesamum indicum* (seed and oil)	Male C57/BL6 mice	Left renal nephrectomy and right kidney ischemia for 30 min followed by 24 h reperfusion	Inhibiting tubular cell death and inflammatory response, upregulating CD39-adenosine-A2AR signals	Oral Pre-treatment	↓SCr, BUN, MDA ↓Caspase-3 expression ↓Infiltration of Ly6G+ neutrophils ↓MPO activity ↓TNF-*α*, IL-1*β* ↑Adenosine level	[161]
Silymarin	*Silybum marianum*	Male Sprague Dawley rat	Right nephrectomy with left renal ischemia for 45 min followed by 6 h reperfusion	Anti-inflammatory, antinecrosis, free radical scavenging		↓Tubular dilatation ↓Tubular vacuolization ↓Inflammation ↓Tubular and glomerular necrosis	[162, 163]
Total flavonoids	*Rosa laevigata*	NRK-52E cells male Sprague Dawley rat	Bilateral renal ischemia for 45 min followed by reperfusion for 24 h	Attenuating oxidative stress and inflammation		↓SCr, BUN, MDA, ↓Levels of Keap 1 and NF-KBP65 ↓IL-1*β*, IL-6, TNF-*α* ↑Sirt 1, Nrf 2, and HO 1 ↑GSH, GPx, SOD	[108]

Ursolic acid	*Crataegus* sp., *Arctostaphylos uva-ursi*, Chinese elder herb, *Actinidia deliciosa, Prunella vulgaris*	Male Sprague Dawley rat	Right renal nephrectomy and left kidney ischemia for 45–90 minutes	Decrease in oxidative stress. Suppressing STAT3, NF-*κ*B and caspase-3 activities.	Posttreatment	↓SCr, Angiotensin II ↓STAT3 protein phosphorylation ↓NF-*κ*B expression Inhibit caspase-3 activity	[164]

## Data Availability

The data used to support the findings of this study are all included and available within the article.

## Abbreviations

AKI: Acute kidney injury
AQP: Aquaporin
AST: Aspartate amino transferase
ATP: Adenosine triphosphate
BUN: Blood urea nitrogen
CKD: Chronic kidney disease
COX-2: Cyclooxygenase-2
eNOS: Endothelial nitric oxide synthase
ESRD: End-stage renal disease
FasL: Fas ligand
FENa+: Fraction sodium excretion
FRAP: Tissue ferric-reducing antioxidant power
GFR: Glomerulus filtration rate
GPx: Glutathione peroxidase
GSH: Glutathione
HIF-1*α*: Hypoxia inducible factor-1 alpha
HMGB1: High mobility group box-1
HO 1: Heme-oxygenase
HSP 70: Heat shock protein 70
I/R: Ischemic reperfusion
ICAM-1: Intercellular adhesion molecule-1
ICU: Intensive care unit
IFN-*ɣ*: Interferon gamma
IGFBP-7: Insulin-like growth factor binding protein 7
IL: Interleukin
iNOS: Inducible nitric oxide synthase
JAK2/STAT3: Janus kinase 2/signal transducer and activator of transcription 3
Keap 1: Kelch-like ECH-associated protein-1
KIM-1: Kidney injury molecule 1
LDH: Lactate dehydrogenase
MCP: Monocyte chemoattractant protein
MDA: Malondialdehyde
MPO: Myeloperoxidase
MPTP: Mitochondrial permeability transition pore
mRNA: Messenger ribonucleic acid
MyD88: Myeloid differentiation primary response gene
NADPH: Nicotinamide adenine dinucleotide phosphate
NF-KBP65: Nuclear translocation of nuclear factor-Bp65
NGAL: Neutrophil gelatinase associated lipocalin
NO: Nitric oxide
Nrf-2: Nuclear factor erythroid 2-related factor-2
NR-F2: Nuclear factor erythroid 2-related factor 2
PI3K/Akt: Phosphatidylinositol-3-kinase
RIRI: Renal ischemic reperfusion injury
RNS: Reactive nitrogen species
ROS: Reactive oxygen species
ROS: Reactive oxygen species
RRT: Risk of renal replacement therapy
SCr: Serum creatinine
Shh: Sonic Hedgehog
Sirt 1: Silent information regulator factor 2-related enzyme 1
SOD: Superoxide dismutase
TBARS: Thiobarbituric acid reactive substances
TIMP-2: Tissue inhibitor of metalloproteinase-2
TLR: Toll-like receptors
TNF: Tissue necrosis factor
TRAP 6: Tumor necrosis factor receptor associated factor 6
VCAM: Vascular cell adhesion molecule.
